# PET and PVC Separation with Hyperspectral Imagery

**DOI:** 10.3390/s150102205

**Published:** 2015-01-20

**Authors:** Monica Moroni, Alessandro Mei, Alessandra Leonardi, Emanuela Lupo, Floriana La Marca

**Affiliations:** 1 DICEA-Sapienza University of Rome, via Eudossiana 18, 00184 Rome, Italy; E-Mail: emanuela.lupo@uniroma1.it; 2 CNR—Institute of Atmospheric Pollution Research, Area della Ricerca di Roma1, Via Salaria Km 29,300 Monterotondo, I-00015 Rome, Italy; E-Mail: mei@iia.cnr.it; 3 DICMA-Sapienza University of Rome, via Eudossiana 18, 00184 Rome, Italy; E-Mails: alelnd@alice.it (A.L.); floriana.lamarca@uniroma1.it (F.L.M.)

**Keywords:** recycling, plastic polymers, hyperspectral imaging, NIR, PET, PVC

## Abstract

Traditional plants for plastic separation in homogeneous products employ material physical properties (for instance density). Due to the small intervals of variability of different polymer properties, the output quality may not be adequate. Sensing technologies based on hyperspectral imaging have been introduced in order to classify materials and to increase the quality of recycled products, which have to comply with specific standards determined by industrial applications. This paper presents the results of the characterization of two different plastic polymers—polyethylene terephthalate (PET) and polyvinyl chloride (PVC)—in different phases of their life cycle (primary raw materials, urban and urban-assimilated waste and secondary raw materials) to show the contribution of hyperspectral sensors in the field of material recycling. This is accomplished via near-infrared (900–1700 nm) reflectance spectra extracted from hyperspectral images acquired with a two-linear-spectrometer apparatus. Results have shown that a rapid and reliable identification of PET and PVC can be achieved by using a simple two near-infrared wavelength operator coupled to an analysis of reflectance spectra. This resulted in 100% classification accuracy. A sensor based on this identification method appears suitable and inexpensive to build and provides the necessary speed and performance required by the recycling industry.

## 1. Introduction

The annual production of plastic wastes in Europe in 2012 was 57 millions of tons; 62.2% of the total derives from household wastes (mainly container and packaging). In the same year, mechanical recycling involved about 26% of total post-consumer plastics, 0.3% was collected for feedstock recycling, and 35.6% for energy in municipal waste incineration plants or as refuse derived fuel material. The residual 38.1% of plastic waste was landfilled. Though a consistent decrease in delivery of plastic materials in landfill has been observed in the last years, improvements in plastic recovery are still needed to reduce the disposal rate down to the goal of zero plastic wastes in landfills by 2020 in Europe [1].

Due to their great chemical stability, degradation of plastics takes a long time and has a significant environmental impact. On the other hand, due to the processes used in their production, comprising moulding or extrusion in many cases, they can be reused with high efficiency [2]. This implies they are separated from the rest of the wastes and classified by type via recycling processes. Recycling requires the separation of materials appearing in a mass of wastes of heterogeneous composition and characteristics, into single, almost pure, component/material flows [3].

A wide variety of polymers can be found in household plastic wastes. Though the largest amounts are represented by polyethylene (LDPE and HDPE, 29.5%), polypropylene (PP, 18.8%) and polyethylene terephthalate (PET, 6.5%); a considerable percentage (about 8%–10%) of polyvinyl chloride (PVC) can also be found.

The low cost of virgin polymers and low value of recycled plastics require the utilization of low cost techniques and processes in the recycling of plastic materials. The application of mechanical separation to plastic wastes may be challenging due to the similarity of their properties [4]. Separation of polymers of density lower than 1 g/cm^3^ from polymers of density higher than 1 g/cm^3^ is quite simple and it is typically achieved via gravimetric methods. The separation of polymers of density higher than 1 g/cm^3^, for instance PET and PVC, is commonly carried out manually or, in few cases, adopting very sophisticated mechanical processes [5–7].

PET (PolyEthylene Terephthalate) is a thermoplastic resin of the polyester family. It is utilized to produce synthetic fibers, beverage and other liquid containers, food packaging, *etc*. PVC (Polyvinyl chloride) is a thermoplastic polymer mainly used in the building and construction sector to produce, for instance, pipes, window frames, cable insulation, floors, coverings, roofing sheets. PET density usually ranges from 1.33 to 1.37 g/cm^3^ whereas PVC density ranges in a larger interval 1.10–1.61 g/cm^3^ which partially overlaps the PET density range.

PVC and PET have been selected among the other plastic polymers because they both have density values larger than 1 g/cm^3^ and generally within a small interval. This means their separation may be challenging with traditional methods. Furthermore, PET melting temperature (250–260 °C) is higher than PVC one (140–160 °C). This implies that during PET processing, PVC degrades producing a residue material which affects the quality of the product, while, on the other hand, PET does not plasticize at PVC processing temperatures and must be removed by filtration [8]. Finally, the presence of PVC in plastic waste may generate environmentally hazardous chlorinated compounds such as gaseous hydrogen chloride and dioxins containing chlorine during melting processes for material recycling and thermal energy recovery [9,10].

Thus, it is necessary to develop effective technologies to separate chlorinated from other plastic polymers. The hyperspectral method represents a valuable methodology alternative to more traditional tools to separate different types of plastic polymers as well as contaminants from plastic wastes. This technology combines spectral reflectance measurements and image processing technologies. It is an effective, low cost, reliable and efficient method that allows overcoming the typical problems of the most used separation methods, such as: the influence of moisture, surface status and low feeding speed of particles in electrostatic separation [11]; the need of additive addition in separation by flotation and density [12–14]; the wide range of density values for the same typology of plastic materials making the choice of the density for sink and float separation challenging [15,16]; the need for more separation steps to classify a heterogeneous mixture of plastic wastes containing different (more than two) useful fractions [17].

The hyperspectral analysis, originally developed for remote sensing applications and military surveillance, is actually successfully employed in many other fields, such as agriculture [18], asphalt characterization [19], healthcare [20] and pharmaceutical [21] sectors. Remarkable is the intensive use of the hyperspectral technology in the food sector as a technique for inspection of a wide range of products investigated, such as fruits and vegetables, meat, fish, eggs and cereals [22]. Recently, several studies have demonstrated that the hyperspectral analysis can be successfully used in the field of solid waste recycling and treatment, with reference to paper [23], glass [24], polymers [25] and polyolefin [26].

Scott [27] described a device for automated sorting of post-consumer PET and PVC wastes. It comprised a two-color fixed filter near-infrared spectrometer and a ratio circuit. No statistical analysis is provided in the article to validate the choice of the two wavelengths employed for the classification. Van den Broek *et al.* [28] presented a two-step procedure for the acquisition of spectroscopic image data and the supervised classification by a neural network of the measured images. The procedure was applied to mixtures of plastic and non-plastic materials with performances below 90% of correct classification. Leitner *et al.* [25] presented a real-time classification of waste polymers in a prototype of an automated industrial sorting facility. Best performance in terms of pixel-wise classification is achieved with the dissimilarity-based classifier, which classified around 93% of the sample spectra correctly. Ulrici *et al.* [29] demonstrated the effectiveness of hyperspectral imaging in the near infrared range in discriminating PET from PLA (poly(lactic acid)), two polymers commonly utilized as packaging for foodstuff. Partial Least Squares-Discriminant Analysis was used to classify three classes, *i.e.*, background, PET and PLA with prediction efficiency in the order of 98%.

The aim of the paper is twofold: on the one hand the spectrometer platform and its setup are presented; on the other one, the flexibility of the platform, employed in the field survey described in [30], was demonstrated with the laboratory investigation of two plastic polymers, *i.e.*, PVC and PET. This paper further presents a spectral signatures' statistical analysis for the individuation of a reliable index suitable for a fast and more efficient classification process of PET and PVC than previous investigations. The samples consist of plastic materials at different stages of their life cycle, namely virgin particles (primary raw materials), wastes coming from different sources (urban and urban-assimilated waste) and regenerated plastics (secondary raw materials). For both typologies of polymer, several samples of different forms, *i.e.*, granules, flakes and pieces, have been analyzed. This should assure that, for the same type of polymer, statistically significant reference spectral signatures have been obtained. Due to the inefficiency of the VISible (VIS) part of the electromagnetic spectrum for polymer differentiation, only the Near InfraRed (NIR) region has been considered. A robust statistical and absorption feature characteristic analysis has been conducted to identify the wavelengths which are the most suitable for the separation of the different plastic polymers.

While several platforms based on NIR sensors for plastic classification are actually available on the market, in most of the cases technical aspects are unknown or covered by trade secret. Commercial facilities mainly treat unprocessed wastes, for instance entire bottles. One of the strengths of this contribution is the investigation of plastic samples of PET and PVC with different shapes and at different stages of their life cycle underlining the flexibility of the proposed procedure.

This paper is organized as follows. Section 2 describes the plastic materials investigated, the hyperspectral device with two spectrometers and the methodology to extract spectral signatures from the images acquired. Section 3 presents the main results in terms of the spectral signatures detected with the platform, the statistical procedure employed to analyze data and the classification performances of the indices. The paper ends with concluding remarks.

## 2. Materials and Methods

### 2.1. Plastic Materials Tested

**Figure 1.**
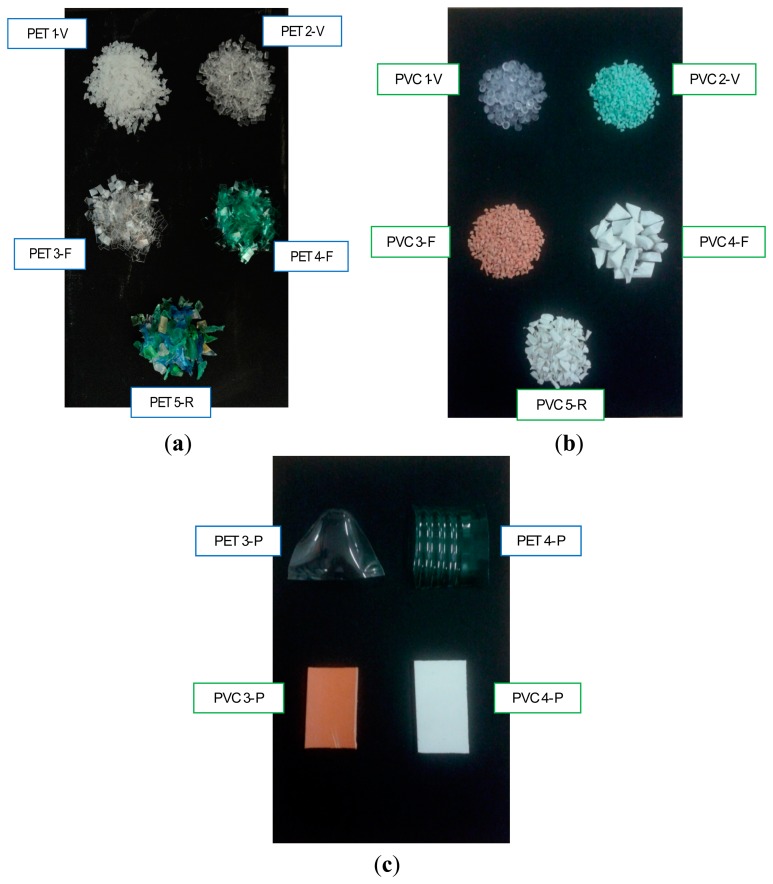
Images of (**a**) virgin, waste and regenerated PET samples; (**b**) virgin, waste and regenerated PVC samples; (**c**) PET and PVC samples in pieces (for the nomenclature refer to Table 1).

Plastic samples of PET and PVC have been collected at different stages of their life cycle (Figure 1). presents the characteristics of the samples in terms of origin, color, density, mean particle size. For each sample, roughly 50 g of material have been analyzed.

Four samples of virgin plastic particles (PET 1-V, PET 2-V, PVC 1-V, PVC 2-V), consisting of nearly spherical or cylindrical granules of different color, density and composition, represent the primary raw materials used for the manufacture of products (Figure 1a,b).

Urban and urban-assimilated plastic wastes have been collected from many sources. To analyze operating conditions more similar to those occurring in a plant, samples of plastic materials in flakes (PET 3-F, PET 4-F, PVC 3-F, PVC 4-F; Figure 1a,b) and in large pieces (PET 3-P, PET 4-P, PVC 3-P, PVC 4-P; Figure 1c) have been selected. This allows the investigation of the influence of the sample geometry on the measured spectral signatures. Each waste sample was washed, purified from any impurities and then size-reduced manually or with a knife mill. As can be seen from Figure 1c, plastic wastes in pieces, unlike previous samples, have irregular shapes and mimic the form of fragments originated from coarse crushing operations.

Finally, two samples (PET 5-R, PVC 5-R, Figure 1a,b) have been selected from two Italian plants for recovery and recycling of plastic materials (Rigenera S.r.l., Terni; “Montello S.p.a.”—Montello (BG)). These materials represent the second raw materials used for the production of new goods and products. Samples present a rather irregular shape as a consequence of the recycling process they have been gone through.

Each sample was characterized by determination of average size (geometric characterization) and density (physical characterization). Table 1 shows the large range of density values characterizing the same polymer, mainly PVC, due to the auxiliary substances used for its transformation. Each density value is the result of the arithmetic average of three independent measurements. The wide range of density assumed by the same polymer further demonstrates how hyperspectral systems represent a viable alternative to traditional systems of separation based on density.

The average size of the samples was determined by a gage. For particles of spherical shape, the average diameter was chosen as the reference size, whereas for particles of rectangular or elongated shape the average of the side dimensions was considered.

To assess the spectral signature in real-plant conditions, all plastic samples have been placed on a dark conveyor belt.

### 2.2. The Platform

#### The hyperspectral platform

At the Laboratory of Hydraulics of DICEA-Sapienza University of Rome, an effective methodology for hyperspectral investigations has been developed. It is based on the use of two experimental devices for acquiring hyperspectral images, one based on the use of tunable interference filters, the other on the use of spectrometers. Both systems allow sampling the spectral range of 400–1700 nm. The system with interference filters has been employed for recognition of vegetation, and detecting diseases and abnormalities in the spectral signatures of plant species [31]. The system with spectrometers, added to a synchronized CMOS 4M60 Dalsa camera (equipped with a standard lens) moving in unison with the device, and an original algorithm for automatically combining multiple, overlapping images of a scene to form a single composition, have been employed in a proximal sensing field campaign conducted in San Teodoro (Olbia-Tempio—Sardinia). Mapping allowed for the identification of objects within the acquired image and agreed well with ground-truth measurements [30].

**Figure 2.**
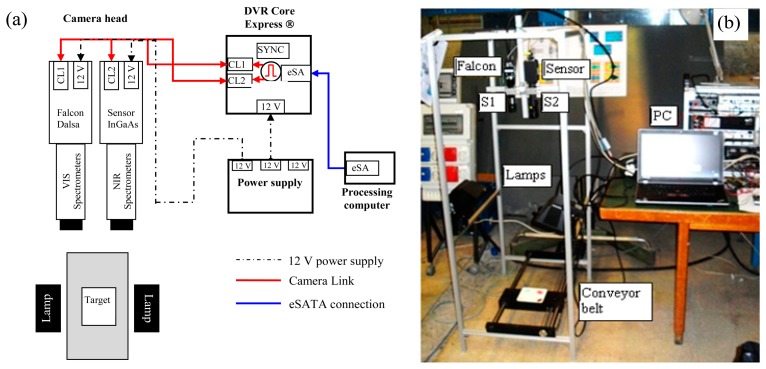
(**a**) Diagram of the hyperspectral device with two spectrometers. CL stands for Camera Link, eST for eSATA connection, SYNC for synchronization signal; (**b**) Sketch of the laboratory facility. S1 stands for VIS spectrometer and S2 for NIR spectrometer.

The system with spectrometers was employed for the experimental investigation of plastic samples. Figure 2 shows a diagram of the system configuration, comprising: one VIS Specim Imspector spectrometer (S1), centered in the visible range of the electromagnetic spectrum (400 nm to 1000 nm) mounted in front of a Dalsa Falcon 1.4M100 CMOS camera of 1400 × 1024 pixel resolution, 7.4 μm × 7.4 μm pixel pitch, 100 fps maximum acquisition frequency; the images presented in this work were acquired at 25 fps and the achieved spectral resolution was 3 nm; one NIR Specim Imspector spectrometer (S2), centered in the near infrared region (900 nm–1700 nm), mounted in front of an InGaAs Sensor Unlimited camera, 320 × 240 pixel resolution, 25 μm × 25 μm pixel pitch, 50 fps maximum frequency of acquisition; the images presented in this work were acquired at 50 fps and the spectral resolution was 3 nm; one high-speed DVR CORE with two Camera Link inputs used to acquire and manage the data, containing 1-terabyte solid state disk array; one power supply for all system devices; one processing computer for controlling the entire system and acquiring images; one lighting system comprising two 500 Watt halogen lamps; one conveyor belt to allow maintaining the target displacement at a constant rate.

#### Spectral calibration

The spectral calibration of the hyperspectral sensor is the procedure required to determine the relationship between the true spectral position of the incoming light and the observed effect. In particular, this operation allows identifying the portion of the sensor useful for the construction of the hyperspectral cube. To ensure the largest portion of the VIS sensor was available for capturing spatial information, the VIS spectrometer slit was set parallel to the sensor rows. For the NIR spectrometers, the 240 rows of the Sensor Unlimited camera were insufficient to acquire the entire spectral set (consisting of 254 lines). For this reason, the NIR spectrometer slit was set parallel to the sensor columns.

To calibrate the spectrometer-camera systems, three tunable interference filters—the first (VIS) tuning from 400 nm to 720 nm with a bandwidth of 10 nm, the second (SNIR) from 650 nm to 1100 nm with a bandwidth of 10 nm, and the third (LNIR) from 900 nm to 1800 nm with a bandwidth of 6 nm—have been employed.

**Figure 3.**
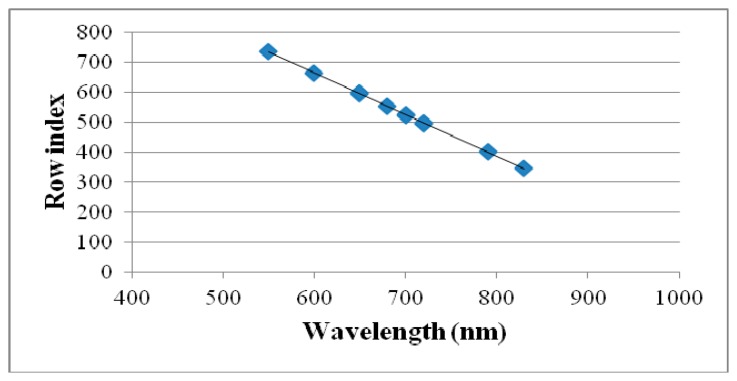
Calibration curve of the VIS spectrometer interfaced with interference filters tuning in the VIS and SNIR regions.

**Figure 4.**
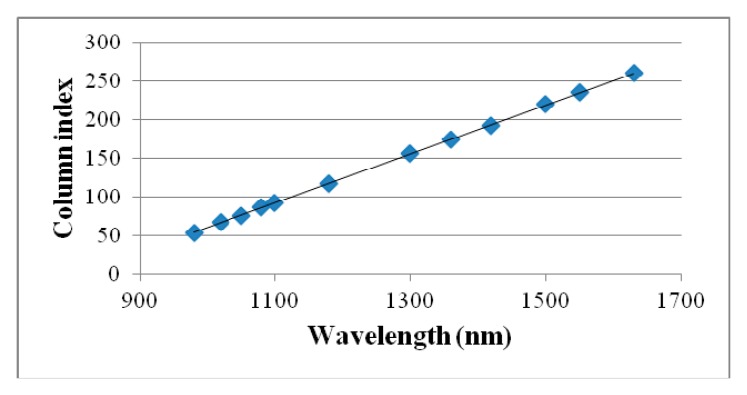
Calibration curve of the NIR spectrometer interfaced with interference filters tuning in the SNIR and LNIR regions.

The calibration procedure consisted in interposing one filter at the time between the proper spectrometer and a reference object. The reference object used to calibrate the system was a Spectralon target illuminated through two halogen lamps. A series of predetermined wavelengths have been imposed to the filter. The known spectral wavelengths of the emission lines (bright foreground of dark background images) have then been regressed against the position of the pixels within the sensor in which the response was recorded. Figures 3 and 4 show how first order regressions properly summarize the spectral data for both the VIS and NIR spectrometers.

The spectral information for the VIS spectrometer is split into 840 rows with a spectral resolution of 1.4 pixel/wavelength. For the NIR spectrometer, the spectral information occupies 254 columns with a spectral resolution of 0.32 pixel/wavelength.

### 2.3. Methodology

Hyperspectral imaging system is based on the utilization of an integrated hardware and software architecture able to digitally capture and handle spectral attributes of each pixel in an image. Thus, a hyperspectral image, namely hypercube, is a three dimensional dataset with two spatial dimensions and one spectral dimension [26]. The hypercube allows the visualization of sample specific attributes, recalling that regions of the sample with similar spectral properties have similar chemical composition.

The characterization of materials via the hyperspectral platform requires the following steps.

#### Imagery acquisition and processing

Linear spectrometer captures a line image of the target and disperses the light from each line image pixel into a spectrum. Each high-resolution spectral image contains then line pixels in a spatial axis and spectral pixels in a spectral axis. The spatial information is along the x axis, while the spectral information is along the y axis. Multiple images must be acquired to reconstruct a two-dimensional scene based on the combination of several lines. This implies either the target or the acquisition apparatus are moved in a controlled fashion. The spectral image sequence is then employed to create one image for each wavelength of interest. Since the objects were placed on a conveyor belt moving at a constant speed in a known direction, the reconstruction of the scene at the various wavelengths was obtained by simply placing side by side rows (or columns) of the acquired image sequence. This implies the slit of the spectrometer was set perpendicular to the direction of movement of the object and the device collimation axis was normal to the conveyor belt plane.

#### Imagery correction

Vignetting effects have been eliminated from each image. This imperfection causes the reduction of brightness at image edges respect to its center. It is due to the camera lens and to the tunable filters placed in front of the camera. This problem was modeled by a cos^4^(α) fall-off in intensity away from the principal point, assuming that the optic axis passes through the image center. Noise filtering was further considered. The CMOS and InGaAs arrays employed for image acquisitions are subject to various sources of noise, including thermal noise, shot noise, and electronic noise in the amplified circuitry. When the image is digitized, it also suffers intensity quantization, usually to 8 bits of resolution. To reduce noise effects, images have been convolved with a Gaussian mask. No appreciable effects, such as blur, have been noticed in the resulting images.

#### Creation of hyperspectral cube

**Figure 5.**
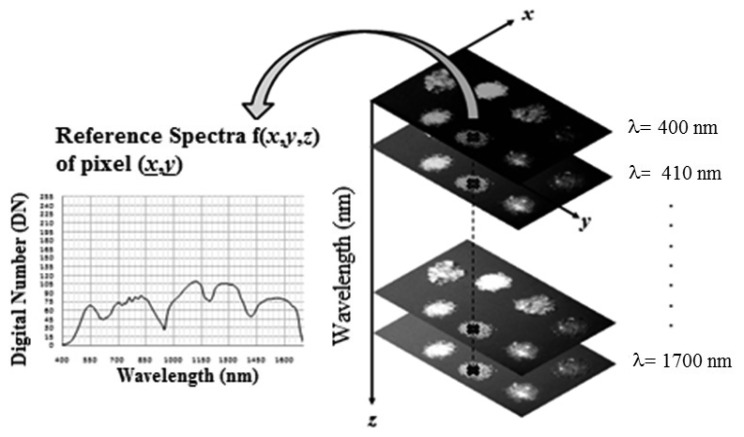
Hyperspectral cube.

It is a three-dimensional array containing spatial information on the x and y axes (image) and spectral information on the z axis (Figure 5).

#### Radiometric calibration

It constitutes one of the most sensitive pre-processing steps, since it ensures the construction of a spectral library as close as possible to the material characteristics. This is achieved by eliminating the dependence on the spectra of the measuring instruments (quantum efficiency of the sensor, filter transmission). In fact, the acquisition system does not record the material reflectance but rather the value of radiance, or that part of the reflected radiation that reaches the camera sensor with energy content sufficient to be recorded. The absolute reflectance of the materials can be calculated only if the incident radiation on the target is known. In this case, with an artificial lamp-light being the source of radiation, it would be impossible to obtain the value of radiation incident on each point of the scene. The relative reflectance is then calculated. This is achieved by comparison with a reference spectrum chosen ad hoc. Two methods can be employed: the Internal Average Relative Reflectance (IARR) and the Flat Field (FF). The first method derives correction parameters directly from the images while the second one requires the presence of targets with smooth reference reflectance spectrum. We have employed the latter method by introducing in the scene a white reference standard. A high-density fluoropolymer panel (Spectralon), assumed as Lambertian surface, is used as white reference to retrieve spectral signatures on reflectance values.

#### Clustering and extraction of spectral signatures

This step is performed on the spectrally and radiometrically calibrated hyperspectral cube to produce an image in which pixels with similar spectral signatures are associated with the same class. This allows defining a spectral library or set of reference spectra. Through the analysis of the spectra, it is possible to recognize, and then classify, the different materials.

#### Statistical analysis

Due to the high spectral resolution of the hyperspectral device, the entire dataset encompasses a large amount of information. To reduce data dimensionality, spectral signatures have been employed to compute the correlation matrix. It allows highlighting dissimilarities between spectral bands and finding, within the entire dataset, low correlation values between wavelength couples. Hence, self-consistent information bands are emphasized and redundant information may be ignored. The correlation matrix has been used by different authors for defect detection on apples [32], for feature extraction from hyperspectral data [33], for inland water quality mapping [34], for geological unmixing classification [35]. Furthermore, spectral signatures have been processed for Continuum Removal (CR). CR normalizes spectra by applying a convex hull over that part of the spectrum that will be analyzed [36]. First, the continuum line is determined by estimating the wavelengths where the reflectance presents high values, assuring absorption wavelengths are not included. Once the continuum line is established, the continuum-removed spectra are calculated by dividing the original reflectance values by the corresponding values of the continuum line. The peak reflectance points are then standardized to a value of one; this value decreases toward zero as the distance between the original spectrum and the continuum line increases. This technique allows emphasizing absorption peaks [37]. CR has demonstrated to be useful to identify the geological composition of materials [38] or for vegetation analysis [39].

## 3. Results and Discussion

In this section, representative spectral signatures and the absorption feature characteristics of both polymers are presented. Statistical analysis for spectral index extraction and validation are provided for data dimensionality reduction. Due to the dependence of spectral signatures on colors, wavelengths in the VIS region were ineffective for differentiating PET and PVC. Consequently, analyses have been restricted to the NIR region.

### 3.1. Analysis of the Spectral Library

From the hyperspectral cube of each plastic sample, Regions of Interest (ROI) have been created, and corresponding signatures have been extracted as spectral library. Due to the heterogeneity in the ROI dimensions, this spectral library was constituted by a different number of signatures: 3229 for PET primary raw material samples, 5634 for PVC primary raw material samples, 3156 for PET waste samples in flakes, 5218 for PVC waste samples in flakes, 2210 for PET waste samples in pieces, 7682 for PVC waste samples in pieces, 1525 for PET secondary raw material samples, 2210 for PVC secondary raw material samples. To establish an optimal and homogeneous dataset for spectral index computation and validation, the initial dataset has been reduced and 1000 spectral signatures for each sample have been randomly extracted from the original ROI. Then, the employed dataset comprises 14.000 spectral signatures. The number of 1000 signatures was chosen by considering the less numerous sample (PET 4-F).

Successively, representative reflectance spectral signatures of both PET and PVC at different life cycle stages, in the range 950–1700 nm, have been computed by consistently averaging the signatures within the spectral library.

**Figure 6.**
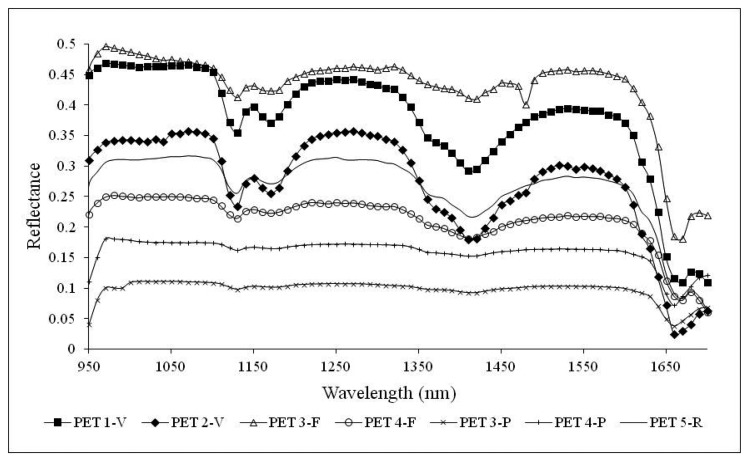
Representative NIR signatures of PET samples on conveyor belt.

Figure 6 shows the NIR signatures for PET samples. No matter the life cycle stage, PET samples present well recognizable characteristic peaks. PET primary and secondary raw material samples show well identifiable absorption peaks at bands 1130 nm, 1170 nm, 1420 nm and 1660 nm. Spectral signatures of waste samples in flakes show evident peaks at 1130 nm and 1660 nm, while at 1170 nm and 1420 nm they are less evident. A remarkable difference can be observed for the spectral signatures of plastic waste samples in pieces (PET 3-P and PET 4-P) which do not exhibit the characteristic peaks shown by the other materials, with the exception of the peak at 1660 nm. This is probably due to the anisotropic reflection of the incident radiation, the support characteristics, the sample thickness and the different light reflection of granular materials respect to pieces. It is worth noting that for PET samples in pieces, a single fragment of material was used for the investigation. Each fragment presented a curvature and it was transparent and thin. Few light reflections then occurred. Furthermore, an influence of the conveyor belt on the measured spectral signatures is expected. In fact, the spectral signatures within the ROI were more heterogeneous than with the other samples. The absorption band at 1660 nm is evident for all life cycle stage materials, while the one at 1170 nm is distinguishable only for primary and secondary raw material samples.

**Figure 7.**
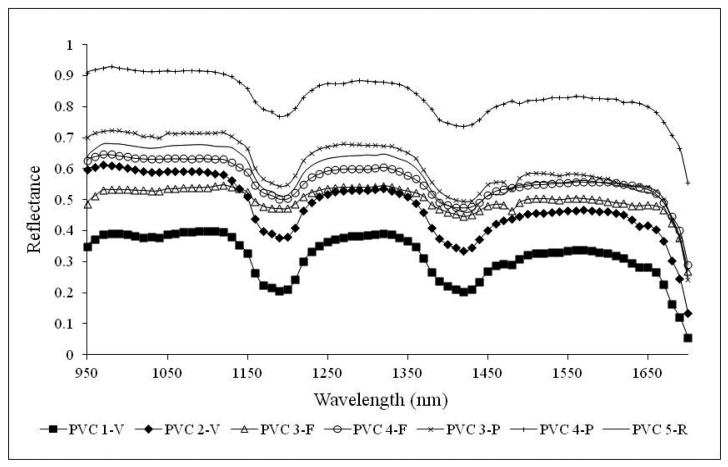
Representative NIR signatures of PVC samples on conveyor belt.

Figure 7 shows the representative NIR signatures for PVC samples. The spectral signatures of PVC primary and secondary raw material samples show two well recognizable characteristic peaks at wavelengths of 1200 nm and 1420 nm. A similar behavior can be observed for waste samples in pieces and flakes. Unlike PET samples, PVC pieces are more opaque and negligibly influenced by the background and for this reason their spectral signatures present the same features as primary raw material samples. Finally, referring to PVC 3-F and PVC 3-P, a small absorption peak is appreciable also at a wavelength of 1480 nm.

Absorption peaks are strictly related to the polymer chemical structure. In the NIR region, the light is absorbed by polymers via the first overtones of the normal modes of vibration involving stretching of the C-H and O-H bonds [27]. As shown by [40,41], PET and PVC may be distinguished by locating the wavelength of C-H stretching. The position of this spectral feature has been determined by measuring the ratio of the absorbance at 1716 nm and at 1660 nm by [27] and analyzing absorbance values at 1656 nm for PET and at 1712 for PVC by [42].

**Figure 8.**
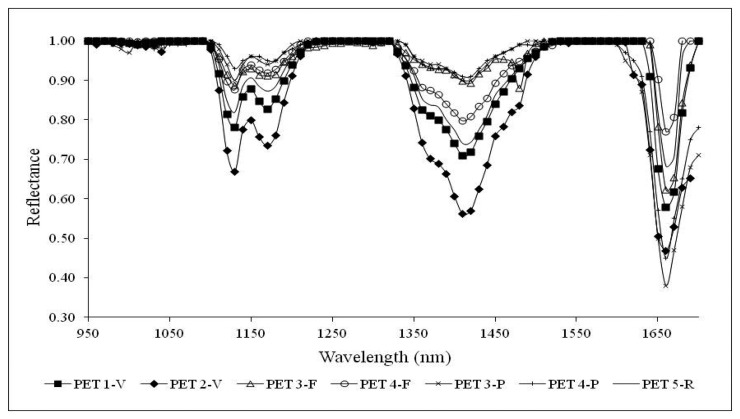
NIR signatures after continuum removal of PET samples on conveyor belt.

**Figure 9.**
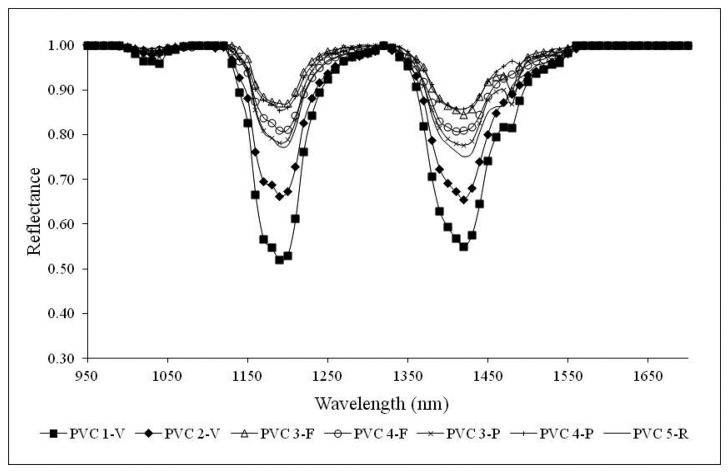
NIR signatures after continuum removal of PVC samples on conveyor belt.

A different approach is presented here. Firstly, to highlight absorption band positions and characteristics, the continuum removal processing has been adopted. Each signature of the spectral library was divided by a reference spectrum with a higher overall reflectance, producing a ratio spectrum which amplifies spectral differences at wavelengths related to the absorption bands [43]. For CR spectrum calculation, wavelengths associated to maxima of reflectance values have been selected to be optimal for both kinds of polymers. These points are located at wavelengths of 970 nm, 1090 nm, 1320 nm and 1580 nm. Figures 8 and 9 present PVC and PET CR curves which highlight the already well identified absorption peaks and other smaller ones.

For PET primary and secondary raw materials and waste samples in flakes and pieces, absorption bands at 1130 nm, 1170 nm, 1420 nm and 1660 nm are well recognizable and definitely emphasized from the CR operation.

Figure 6 averages out the effects of anisotropic reflection of the incident radiation, support characteristics and the sample thickness as demonstrated in Figure 8 where the characteristic peaks of PET are enhanced also for PET 3-P and PET 4-P. Absorption peaks of PVC samples at 1190 nm and 1420 nm are accentuated as well as the peak at 1480 nm for PVC 3-F and PVC 3-P.

The absorption band positions and depths have been calculated from the continuum removal spectra. The band depth at a given wavelength (λ_i_) is computed by subtracting the Continuum Removal Reflectance (CRR) from 1 [36]:

(1) $$\text{BD}\;(\lambda_{\text{i}})=1-\text{CRR}\;(\lambda_{\text{i}})$$

Table 2 shows the results of band position and depth computation.

Most prominent absorption band depths of primary raw material sample spectral signatures are located at 1660 nm (0.460) for PET and at 1200 nm (0.396) for PVC. PET waste samples in flakes and pieces show higher band depth at 1660 nm (respectively 0.308 and 0.585) while regenerated polymers at 1420 nm (0.216). At the same time, PVC waste samples in flakes and regenerated materials show higher values at 1420 (respectively 0.176 and 0.252) while PVC wastes in pieces at 1200 nm (0.219), analogously to raw material samples.

### 3.2. Reduction of Data Dimensionality and Spectral Index Retrieval

Unique features of each polymer typology may be gathered by measuring the absorption of light at a few well-selected wavelengths. Two strategies are adopted for spectral index retrieval: the first based on absorption band position and depth and the second on the correlation matrix analysis.

#### 3.2.1. Analysis of Absorption Feature Characteristics

The analysis of both reflectance and continuum removal spectral signatures has allowed highlighting the characteristic absorption peaks. Their combination (ratio and difference) should then be adequate to differentiate PET and PVC. PET spectral signatures show two well distinguishable absorption peaks at 1130 nm and 1660 nm. Hence, two spectral indices (1130–1660 and 1130/1660) have been considered (Table 2). In addition, both original and CR reflectance signatures present a reflectance peak at 1150 nm. While PVC signatures highlight a constant diminution of reflectance values between 1130 and 1170 nm, PET samples present a doublet positioned at 1130–1170 nm which exhibits a characteristic trend and curve shape. Based on this shape feature, two further spectral indices (1150–1130 and 1150/1130) have been considered.

Table 3 lists the spectral indices described above. Those ratios and differences have been computed employing the primary raw material dataset and basic statistical quantities evaluated for PET and PVC samples separately. Average values and standard deviations for PET and PVC samples have been reported in Table 3, as well as the difference between the two averages. In addition, two threshold values are proposed for the differentiation of PET and PVC. The first threshold is calculated as the mean of the average values for PET and PVC, the second one considering the mean value of the averages plus and minus their standard deviation. In other words, for the latter threshold, the average with the lower value was summed to the standard deviation, the average with the higher value was subtracted to the standard deviation and difference among those quantities computed.

Though the ratio of reflectance values at 1130 nm and 1660 nm determines the largest difference between averages (2.238), PET sample standard deviation shows a high value (1.703) limiting the effectiveness of that index for differentiating PET and PVC. Difference indices present modest standard deviations but the averages are very similar.

The same analysis was conducted on spectral signatures after continuum removal. The differences between averages are smaller, as smaller are the standard deviations for both PET and PVC samples.

It is worth recalling the PET absorption peak at 1660 nm, measured on spectral signatures after continuum removal, is always well recognizable and higher than 0 while PVC values are always equal to 1. This suggests another spectral index can be computed employing CR spectral signatures and only one spectral band:

(2) $${\text{I}}_{\text{CRR}}=1-\text{CCR}\;(1660\;\text{nm})$$

#### 3.2.2. Correlation Matrix Analysis

The computation and analysis of correlation matrices allow reducing the dataset dimensionality by providing a set of self-consisting bands and ignoring redundant and useless information for PVC and PET differentiation. This in turn allows determining the wavelengths for the better separation between PET and PVC. From the hyperspectral cubes of the plastics under investigation, the spectral signatures of 1000 (per typology) pixels randomly extracted within the ROI occupied by primary raw materials have been considered. The correlation matrix, C, was computed as follows:

(3) $$\text{C}(\lambda_{\text{i}},\lambda_{\text{j}})=\sum_{\text{N}\;\_\;\text{sam}}\frac{\left(\rho (\lambda_{\text{i}})-\bar{\rho (\lambda_{\text{i}})})(\rho (\lambda_{\text{j}})-\bar{\rho (\lambda_{\text{j}})}\right)}{\sigma_{\rho (\lambda_{\text{i}})}\;\sigma_{\rho (\lambda_{\text{j}})}}$$

where ρ(λ_k_) is the reflectance at the generic wavelength λ_k_, $\bar{\rho (\lambda_{\text{k}})}$ is the average reflectance at λ_k_, σ_ρ(λ_k_)_ is the standard deviation of the reflectance at λ_k_ and N_sam is the number of spectral signatures employed to compute the correlation matrix.

As expected, the correlation matrix is comprised between 0 and 1, where the lower values identify couples of wavelengths associated to a low correlation of the reflectance values. Those wavelengths may be combined in a spectral index which will likely be effective in separating PVC and PET.

**Figure 10.**
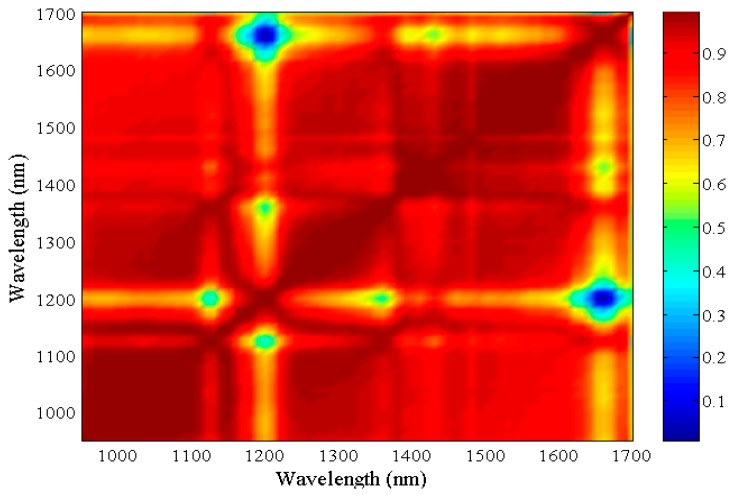
Correlation matrix of PET and PVC primary raw material samples.

Figure 10 shows the 2-D correlation matrix (computed with an original script in MATLAB). The elements of the matrix for each pair of wavelengths correspond to the R^2^ value of PET and PVC spectral signatures. Blue color areas highlight four minimum correlation values: R^2^ = 0.02 at wavelengths 1660 nm *vs.* 1200 nm; R^2^ = 0.41 at wavelengths 1200 nm *vs.* 1130 nm; R^2^ = 0.477 at wavelengths 1200 nm *vs.* 1360 nm and R^2^ = 0.52 at wavelength at 1420 nm *vs.* 1660 nm. For each of these band combinations, ratio and difference indices have been computed and results are presented in Table 4.

For spectral index validation, only wavelength combinations which present low standard deviation values are taken into account. Most prominent differences between PET and PVC averages are reached for the ratios and differences of reflectance values at 1660 nm and 1200 nm and 1200 nm and 1130 nm. It is worth noting this occurs for both original and continuum removal reflectance. In both cases, indices 1200–1130, 1200–1360 and 1420–1660 show positive values for PET and negative for PVC while the opposite occurs for 1660–1200.

### 3.3. Training and Validation of Spectral Indices

**Figure 11.**
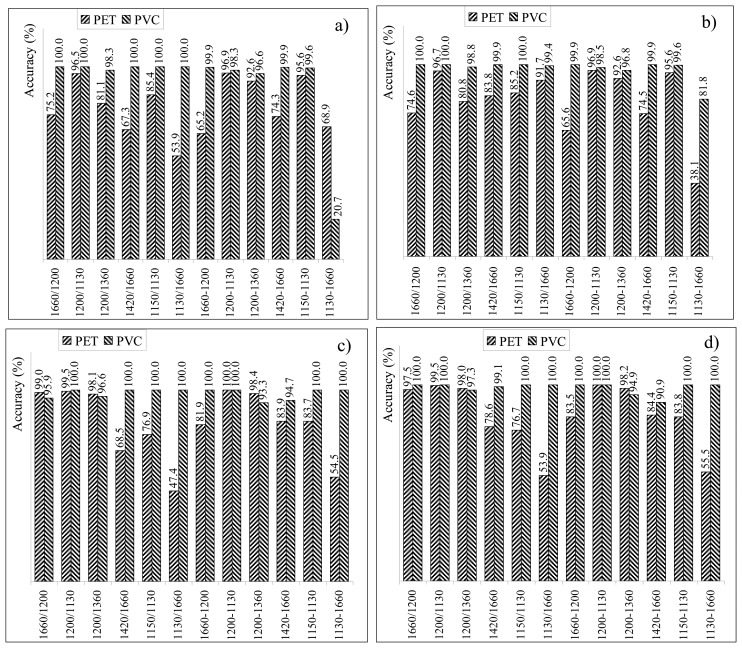
Accuracy assessment of spectral indices for (**a**) original reflectance values and threshold as average of averages; (**b**) original reflectance values and threshold as average with standard deviations; (**c**) CR reflectance values and threshold as average of averages; (**d**) CR reflectance values and threshold as average with standard deviations.

The threshold values have been identified processing primary raw material spectral signatures. Hence, for virgin materials, the accuracy of the classification process is 100% (where the accuracy is defined as the percentage of properly assigning a certain spectral signature to the proper category, *i.e.*, PET or PVC). The accuracy evaluation step is then mandatory to verify if those thresholds are suitable to classify all polymer samples, no matter their life cycle stage. Hence, the entire dataset was employed for the accuracy step. For each index, each spectral signature of the dataset has been employed to compute the ratio or difference among the reflectance values and the result compared with both thresholds. According to the result of the comparison, the spectral signature has been attributed to a certain typology of plastics. If the assignment is the proper one, the accuracy term for that plastic typology is increased by one. The accuracy terms for both PET and PVC are then normalized with the total number of samples belonging to the dataset (*i.e.*, 7000 samples for both PET and PVC). Furthermore, both original and CR reflectance values have been employed for index accuracy evaluation. Figure 11 provides accuracy assessment results.

Accuracies range between 68.9% for PET and 20.7% for PVC, for the index computed as the difference of reflectance values at 1130 nm and 1660 nm (with threshold set at the average of averages), and 99.5% (100.0%) for PET and 100.0% (100.0%) for PVC, for the ratio (difference) index computed with reflectance values at 1200 nm and 1130 nm and CR values and both threshold values.

Figure 11 highlights two aspects: (1) a simple operation as Continuum Removal improves the overall classification results; (2) the choice of the threshold is not critical, assuming it is within the interval comprised between the averages. The classification performances for PVC are remarkable for most of the cases.

The accuracy for indices employing CR reflectance values is overall higher than the corresponding one when original data are used.

Though it employs only the wavelength at 1660 nm, I_CRR_ show remarkable accuracy values (97.0% for PET and 100% for PVC).

## 4. Conclusions

The results show that the hyperspectral analysis is suitable to be used to identify, and then separate, PET and PVC. Though reflectance values depend on many factors such as the characteristics and the thickness of the materials, the lighting conditions, the characteristics of the instrumentation used, and the background, different materials are characterized by distinguishing profiles.

One of the main advantages of the hyperspectral analysis is the rapidity of material recognition which allows increasing the volume of samples processed per unit time. Another important advantage is that the procedure requires modest or no preparation of the material (eventually shredding) before recognition.

Plastic samples of PET and PVC have been collected at different stages of their life cycle and corresponding spectral signatures acquired by the use of a hyperspectral platform in the range of 900 nm–1700 nm. Each measurement was subject to an image correction using dark current and reference images. This correction entails the subtraction of the dark current contribution from both the reference and raw image signals followed by a division of the raw image by its corresponding reference image.

PVC reveals three important absorption peaks at wavelengths 1200 nm, 1420 nm and 1480 nm while PET has four peaks at 1130 nm, 1170 nm, 1420 nm and 1660 nm. By the use of these wavelengths, ratio and difference spectral indices have been computed for both reflectance and continuum removal data showing high accuracy values in terms of separation efficacy. In fact, best accuracies (100%) for PET and PVC differentiation are reached using indices 1200 nm/1130 nm and 1200–1130 nm computed from reflectance and CR data.

The hyperspectral analysis conducted in the near infrared region has highlighted that materials belonging to the same type of polymer with similar spectral curves and density can only be differentiated by the use of some wavelengths. This behavior characterizes samples belonging to given plastic typologies no matter the dimension, the phases in the product life cycle (virgin, recovered or post-consumer), or, finally, the form (flakes or pieces). This confirms the validity of hyperspectral imaging for PET and PVC separation, which can be used in any stage of the life cycle of a product.

Nevertheless, the acquisition of reliable and usable spectral signatures for samples in pieces was quite a challenging task. Due to the shape and transparency of PET samples, some of the spectral signatures may be influenced by reflections on the sample surface or from the background (conveyor belt). The latter effect could have been avoided by superimposing several pieces of the same typology. However, in a real recycling plant, any superposition among plastic samples should be avoided. In spite of all this difficulties, the combination of statistical correlation analysis and shape analysis allows to identify some spectral indices able to differentiate such kinds of plastic with simple calculations and in a short amount of time.

## Figures and Tables

**Table 1.** List of PET and PVC samples investigated.

**Name**	**Description**	**Sample Origin**	**Color**	**Measured Density (g/cm^3^)**	**Mean Particle Size (cm)**
PET 1-V	Virgin particles in granules	Primary raw material	White/transparent	1.30	0.20
PET 2-V	Virgin particles in granules	Primary raw material	Transparent	1.31	0.28
PET 3-F	Coca-Cola bottle flakes	Wastes in flakes	Transparent	1.35	0.43 × 0.52
PET 4-F	Water bottle flakes	Wastes in flakes	Green/transparent	1.35	0.36 × 0.72
PET 3-P	Coca-Cola bottle piece	Wastes in pieces	Transparent	1.35	4.07 × 3.31
PET 4-P	Water bottle piece	Wastes in pieces	Green/transparent	1.35	4.81 × 3.71
PET 5-R	Bottle flakes	Secondary raw material (regenerated) in flakes	Multicolor/transparent	1.33	0.64 × 1.02
PVC 1-V	Virgin particles in granules	Primary raw material	Transparent	1.30	0.40
PVC 2-V	Virgin particles in granules	Primary raw material (virgin)	Green	1.37	0.18
PVC 3-F	Tube flakes	Wastes in flakes	Orange	1.61	0.17 × 0.28
PVC 4-F	Processing waste flakes	Wastes in flakes	White	1.61	0.40 × 0.61
PVC 3-P	Tube piece	Wastes in pieces	Orange	1.61	2.47 × 4.97
PVC 4-P	Processing waste piece	Wastes in pieces	White	1.61	3.16 × 4.88
PVC 5-R	Recovered from waste flakes	Secondary raw material (regenerated) in flakes	White	1.44	0.36 × 0.62

**Table 2:** Absorption band positions and depths (from CR signatures).

**Samples (Name)**	**Absorption Band Wavelength (nm)**	**Absorption Band Reflectance Value**	**Standard Deviation**	**Band Depth**
**PET 1-V** **PET 2-V**	1130	0.736	0.064	0.264
	1170	0.790	0.053	0.210
	1420	0.651	0.082	0.349
	***1660***	0.540	0.116	***0.460***

**PVC 1-V**	***1200***	0.604	0.081	***0.396***
**PVC 2-V**	1420	0.606	0.062	0.394

**PET 3-F** **PET 4-F**	1130	0.830	0.043	0.170
	1170	0.880	0.032	0.120
	1420	0.763	0.067	0.237
	***1660***	0.692	0.141	***0.308***

**PVC 3-F** **PVC 4-F**	1200	0.836	0.0422	0.164
	***1420***	0.824	0.320	***0.176***
	1480	0.918	0.073	0.082

**PET 5-R**	1130	0.860	0.044	0.140
	***1420***	0.784	0.050	***0.216***
	1660	0.791	0.167	0.210

**PVC 5-R**	1200	0.773	0.041	0.227
	***1420***	0.748	0.040	***0.252***

**PET 3-P** **PET 4-P**	1130	0.915	0.036	0.085
	1170	0.915	0.034	0.085
	1420	0.905	0.060	0.095
	***1660***	0.415	0.044	***0.585***

**PVC 3-P**	***1200***	0.831	0.036	***0.219***
**PVC 4-P**	1420	0.825	0.040	0.143

**Table 3.** First set of spectral indices. λ_1_/λ_2_ (λ_1_ − λ_2_) implies the computation of the ratio (difference) among reflectance values at wavelengths λ_1_ and λ_2_.

**Measured Reflectance of Primary Raw Materials**							

**Spectral Indices**	**Average PET**	**Standard Deviation PET**	**Average PVC**	**Standard Deviation PVC**	**Difference between Averages**	**Threshold as Average of Averages**	**Threshold as Average with Standard Deviations**
**λ_1_/λ_2_**							
**1150/1130**	1.148	0.002	0.884	0.001	0.265	1.016	1.017
**1130/1660**	3.656	1.703	1.418	0.033	2.238	2.537	0.867
**λ_1_ − λ_2_**							
**1150−1130**	0.043	0.001	−0.053	0.001	0.096	−0.005	−0.005
**1130−1660**	0.214	0.002	0.137	0.003	0.078	0.176	0.176

**Continuum Removal Reflectance of Primary Raw Materials**							

**λ_1_/λ_2_**							
**1150/1130**	1.154	0.002	0.888	0.001	0.2663	1.021	1.022
**1130/1660**	1.477	0.0789	0.964	0.001	0.454	1.191	1.112
**λ_1_ − λ_2_**							
**1150**–**1130**	0.110	0.001	−0.108	0.001	0.219	0.001	0.001
**1130−1660**	0.196	0.008	−0.036	0.001	0.232	0.080	0.072

**I_CRR_**							

**1** − λ**_1660nm_**	0.754	0.098	0.000	0.000	0.754	0.377	0.328

**Table 4.** Second set of spectral indices.

**Measured Reflectance of Primary Raw Materials**							

**Spectral Indices**	**Average PET**	**Standard Deviation PET**	**Average PVC**	**Standard Deviation PVC**	**Difference between Averages**	**Threshold as Average of Averages**	**Threshold as Average with Standard Deviations**
**λ_1_/λ_2_**							
**1660/1200**	0.242	0.005	1.177	0.039	0.935	0.710	0.676
**1200/1130**	1.246	0.009	0.616	0.005	0.630	0.931	0.927
**1200/1360**	1.236	0.005	0.693	0.010	0.5424	0.965	0.969
**1420/1660**	2.913	0.751	0.798	0.005	2.115	1.856	1.109
**λ_1_ − λ_2_**							
**1660−1200**	−0.285	0.0024	0.039	0.001	0.3239	−0.1227	−0.122
**1200−1130**	0.070	0.0003	−0.176	0.001	0.2462	−0.0530	−0.052
**1200−1360**	0.071	0.0003	−0.122	0.001	0.1929	−0.0259	−0.025
**1420−1660**	0.157	0.0012	−0.0660	0.000	0.2230	0.0456	0.045

**Continuum removal reflectance of primary raw materials**							

**λ_1_/λ_2_**							
**1660/1200**	0.581	0.014	1.685	0.056	1.104	1.133	1.091
**1200/1130**	1.268	0.008	0.627	0.006	0.641	0.948	0.946
**1200/1360**	1.174	0.003	0.657	0.006	0.517	0.915	0.918
**1420/1660**	1.263	0.055	0.606	0.004	0.657	0.934	0.883
**λ_1_ − λ_2_**							
**1660−1200**	−0.388	0.011	0.395	0.006	0.783	0.004	0.009
**1200−1130**	0.192	0.002	−0.359	0.005	0.551	−0.084	−0.080
**1200−1360**	0.135	0.001	−0.315	0.005	0.450	−0.090	−0.086
**1420−1660**	0.119	0.009	−0.394	0.004	0.513	−0.137	−0.142
